# In vitro study: methylene blue-based antibacterial photodynamic inactivation of *Pseudomonas aeruginosa*

**DOI:** 10.1007/s00253-024-13009-5

**Published:** 2024-01-23

**Authors:** Laiq Zada, Shahzad Anwar, Sana Imtiaz, Muhammad Saleem, Aamer Ali Shah

**Affiliations:** 1 Applied Environmental and Geo-Microbiology Lab, Department of Microbiology, Quaid-i-Azam University, Islamabad, 45320 Pakistan; 2 Agri & Biophotonics Laboratory, National Institute of Lasers and Optronics College, Pakistan Institute of Engineering and Applied Sciences, Nilore, Islamabad, 45650 Pakistan

**Keywords:** Diode laser, aPDT, Methylene blue, *Pseudomonas aeruginosa*, Photosensitizer, Antibiotics

## Abstract

**Abstract:**

*Pseudomonas aeruginosa* is one of the most antibiotic-resistant and opportunistic pathogens in immunocompromised and debilitated patients. It is considered the cause of most severe skin infections and is frequently found in hospital burn units. Due to its high antibiotic resistance, eliminating *P. aeruginosa* from skin infections is quite challenging. Therefore, this study aims to assess the novel *in vitro* antibacterial activity of methylene blue using a 635-nm diode laser to determine the effective power and energy densities for inhibition of *P. aeruginosa*. The strain was treated with various concentrations of methylene blue and 635-nm diode laser at powers of 300 mW/cm^2^ and 250 mW/cm^2^. The diode laser’s potency in the photo-destruction of methylene blue and its degradation through *P. aeruginosa* were also evaluated. Colony-forming unit (CFU)/ml, fluorescence spectroscopy, optical density, and confocal microscopy were used to measure the bacterial killing effect. As a result, the significant decrease of *P. aeruginosa* was 2.15-log_10_, 2.71-log_10_, and 3.48-log_10_ at 60, 75, and 90 J/cm^2^ after excitation of MB for 240, 300, and 360 s at a power of 250 mW/cm^2^, respectively. However, a maximum decrease in CFU was observed by 2.54-log_10_ at 72 J/cm^2^ and 4.32-log_10_ at 90 and 108 J/cm^2^ after 300 mW/cm^2^ of irradiation. Fluorescence images confirmed the elimination of bacteria and showed a high degree of photo-destruction compared to treatment with methylene blue and light alone. In conclusion, MB-induced aPDT demonstrated high efficacy, which could be a potential approach against drug-resistant pathogenic bacteria.

**Key points:**

*• Combination of methylene blue with 635-nm diode laser for antibacterial activity.*

*• Methylene blue photosensitizer is employed as an alternative to antibiotics.*

*• aPDT showed promising antibacterial activity against Pseudomonas aeruginosa.*

**Supplementary Information:**

The online version contains supplementary material available at 10.1007/s00253-024-13009-5.

## Introduction

Antimicrobial resistance is a global public health concern that contributes to the high risk of infectious diseases and mortalities and has a significant economic impact (Gajic et al. 2022). The extensive use of antimicrobials in both animals and humans, poor strategies for controlling hospital-acquired infections, and a lack of good decontamination and sanitization practices are some of the factors contributing to the rise in AMR in recent decades (Álvarez-Martínez et al. 2020). Moreover, it is reported that antibiotic resistance in bacteria annually imputed about 700,000 mortalities. If preventive and control measures are not implemented, then by 2050, this figure might increase to 10 million deaths per year, with a 100 trillion USD loss to the global economy, which will significantly impact lower-middle-income countries (O'neill 2014; Christaki et al. 2020). The leading causes of severe infectious diseases and mortalities in immunocompromised debilitated elders are *Staphylococcus aureus*, *Klebsiella pneumoniae*, *Acinetobacter baumannii*, *Pseudomonas aeruginosa*, *Enterobacter*, and *E. faecalis* species (Murray et al. 2022)*.*

Especially, *Pseudomonas aeruginosa* is a common opportunistic (gram-negative) pathogen widely distributed in every habitat and frequently linked to severe infections, whereas 10% of all infections acquired in hospitals are attributed to this pathogenic strain. *P. aeruginosa* inhabits moist areas and is thus present in many medical facilities, particularly in chronic wounds, nebulizers, or UTI devices, where the development of biofilms increases the risk of persistency and resistance to antimicrobial drugs (De Oliveira et al. 2020; Soonthornsit et al. 2023). The CDC has classified *P. aeruginosa* as a major concern for the last 10 years due to its contribution of approximately 32,600 morbidities and 2700 mortalities with 767 million USD in annual health expenses (Kunz Coyne et al. 2022). Several resistance strategies make *P. aeruginosa* a significant pathogen that is challenging to treat because it is resistant to numerous types of antimicrobial drugs (Pang et al. 2019). As a result of high resistance to antimicrobial drugs, there is an urgent need for the emergence of novel therapies.

Antimicrobial photodynamic therapy (aPDT) is a potential alternative treatment for infectious diseases caused by pathogenic bacteria resistant to antibiotics. In addition to being effective against a broad range of microorganisms, aPDT also has several advantages, including high selectivity with rapid action as compared to conventional antimicrobial drugs; resistance is improbable; it has the efficacy to eliminate biofilm; and it is relatively cost-effective (Rezaie et al. 2018; Lan et al. 2019; Negri et al. 2023). In aPDT, the ground-state non-toxic photosensitizer (PS) is activated by a specific range of light irradiation, which transfers energy to reactive oxygen species (ROS) and generates toxic singlet oxygen (1O2) that eliminates all drug-resistant bacteria by the oxidation process (Ishiwata et al. 2021; Zhang et al. 2022). Many photosensitizers, including phenothiazines, curcumin, hematoporphyrin derivatives, phthalocyanine, xanthene, and chlorins, have been studied in recent years to improve the efficiency of aPDT (Figueiredo-godoi et al. 2022). Especially one of the most researched photosensitizers, methylene blue (MB), a cationic phenothiazine derivative readily accessible today, has a substantial absorption in red light at a wavelength between 600 and 700 nm which can penetrate in tissue from 0.5 to 1.5 cm absorption and can cause cell death, necrosis, or apoptosis in tissue. There have been a number of clinical experiments on *in vitro* and infected animal models that demonstrated the efficiency of MB against a broad range of microorganisms (Vecchio et al. 2015; Karner et al. 2020).

In this study, we investigated the efficiency of novel aPDT treatment by using a 635-nm red-spectrum diode laser in combination with various concentrations of methylene blue through the *in vitro* inactivation of multi-drug-resistant *Pseudomonas aeruginosa which are involved in severe wounds and skin infections*. The potency of aPDT was recorded using CFU/ml, optical density, fluorescence spectroscopy, and confocal microscopic analysis. The efficacy of MB and diode laser alone has also been evaluated to demonstrate their solo antibacterial activity.

## Material and methods

### Materials

Methylene blue (MB) photosensitizer in powder form was purchased from VWR International BVBA (Leuven, Belgium). In addition, Mueller-Hinton agar (MHA), nutrient broth, and nutrient agar powder were purchased from Oxoid Ltd. (Basingstoke, Hants, UK). The following classes of meropenem, gentamicin, fosfomycin, colistin, chloramphenicol, doxycycline, cefazolin, and nitrofurantoin antibiotic discs were purchased from Liofilchem® (S.r.I. Roseto, Italy). All other chemicals and reagents were obtained from Sigma-Aldrich (St. Louis, USA).

### Methods

#### Bacterial growth and culture condition

A gram-negative ATCC 27853 strain of *Pseudomonas aeruginosa* was provided by the Applied, Environmental, and Geo-Microbiology Lab, Quaid-i-Azam University, Islamabad, and was used to check the *in vitro* efficiency of aPDT.

For reactivation, the strain was streaked from −80 °C stored glycerol stock on a nutrient agar-containing plate and incubated for 24 h at 37 °C. After incubation, the colony from the plate was inoculated into the nutrient broth and incubated overnight at 37 °C in a New Brunswick (INNOVA 43 USA) shaker at 144 rpm under aerobic conditions (Fig. S1 (i&ii)). An aliquot of 5 ml of an overnight incubated bacterial culture suspension was centrifuged (HERMLE Labortechnik Z 366 K, Germany) in a 15-ml tube at 5000 rpm for 10 min (15 °C). The supernatant was discarded, and the bacterial strain was washed three times with PBS and resuspended in sterile PBS. The strain concentration was measured using a microplate spectrophotometer (LTECK Co., Ltd., INNO™ & INNO-M™, Korea) to the optical density of 0.8 at 600 nm. The adjusted bacterial cell to 10^8^ CFU/ml (OD = 0.8) was stored at −20 °C until further use for the *in vitro* experimentation.

#### Antibiotic sensitivity testing

From the revived bacterial culture plate, 3–4 colonies were picked and transferred to 2 ml of (0.9%) normal saline to develop a visual turbidity equal to the 0.5 McFarland standard with a 10^8^ CFU/ml concentration. The bacterial inoculum from normal saline was repeatedly spread on MHA with a sterile swab, while the disc diffusion method was used to confirm the antibacterial activity according to the standards of CLSI 2020 (Clinical and Laboratory Standards Institute 2020). The halos of inhibition were calculated in mm around the antibiotic disc after overnight incubation at 37 °C.

#### Photosensitizer preparation

The powder form of methylene blue (VWR International BVBA, Leuven, Belgium) as a photosensitizer was used for the preparation of a 10 mg/ml stock solution, which was further diluted in phosphate-buffered saline (PBS), and all experiments were run using concentration ranges of 500 μg/ml, 250 μg/ml, 125 μg/ml, 62.5 μg/ml, 31.25 μg/ml, and 15.625 μg/ml (Fig. S2). Until further use, all the concentrations were stored at 4 °C in the dark.

#### Light source for aPDT

A diode laser of 635 nm developed by the National Institute of Laser and Optronics College of the Pakistan Institute of Engineering and Applied Sciences, Islamabad (Fig. 2D), was utilized as a light source for aPDT against *P. aeruginosa* in combination with methylene blue.

The irradiation time was calculated by using Eqs. 1 and 2 (Parasuraman et al. 2020):

1 $$\textrm{Fluence}\ \textrm{energy}\ \left(\textrm{J}/{\textrm{cm}}^2\right)=\textrm{Power}\ \textrm{density}\ \textrm{of}\ \left(\textrm{PD}=\textrm{W}/{\textrm{cm}}^2\right)\times \textrm{T}\ \left(\textrm{seconds}\right)$$

2 $$\textrm{Whereas}\ \textrm{Power}\ \textrm{density}\ \left(\textrm{PD}\right)=\frac{\textrm{output}\ \textrm{power}\ \left(\textrm{mW}\right)}{\textrm{area}\ \left({\textrm{cm}}^2\right)}$$

The device was used in this experiment in sterile conditions for photosensitization with output powers of 300 mW/cm^2^ in the energy 18, 36, 54, 72, 90, and 108 J/cm^2^ and of 250 mW/cm^2^ in the energy 15, 30, 45, 60, 75, and 90 J/cm^2^ at wavelength of 635 nm for exposure time of 60, 120, 180, 240, 300, and 360 s. The diameter of the diode laser beam was 4 mm, and the distance was set between the laser’s tip and the bottom of a 96-well plate at 5 cm. The optical power meter (1918-C Hand-Held Optical Meter, Newport, Irvine, California 92606) was employed to calibrate and measure the power of the diode laser in the whole experiment, positioned 2 cm apart from the glass plate’s surface.

#### Methylene blue photobleaching

A photobleaching experiment was performed using methylene blue to check the efficiency of the diode laser on dye degradation. A 200 μl of MB aliquot from each concentration was transferred into each well of a 96-well plate and exposed to a power of 300 mW/cm^2^. The degradation of methylene blue was visually observed and determined by the reduction in optical density measured by a microplate spectrophotometer (LTECK Co., Ltd., INNO™ & INNO-M™, Korea) at a wavelength of 600 nm. Three measurements were taken after each irradiation for the confirmation of data variation.

#### Biodegradation of methylene blue

Methylene blue was employed to evaluate the tendency of *Pseudomonas aeruginosa* for biodegradation at optimal temperature, condition, and incubation time. The bacterial strain was transferred to each 5-ml nutrient broth-containing test tube. In addition, 1 ml from each concentration of MB was mixed thoroughly in each test tube containing *P. aeruginosa*. After overnight incubation, the biodegradation was observed visually and compared with the control group.

### Antibacterial effect of methylene blue without laser light

The inoculum of *P. aeruginosa* was incubated with each concentration of methylene blue for 20 min at room temperature in the dark to analyze the antibacterial activity of MB in the absence of photosensitization (+MB-L). Ten microliters of bacterial strain was spread on nutrient agar-containing plates, and growth was examined after overnight incubation at 37 °C.

The well diffusion technique was also performed for the efficiency of MB without irradiation. Bacterial colonies were diluted in 0.9% normal saline to equal the turbidity with the 0.5 McFarland standard. Bacterial lawn was prepared on MHA surface plates by using a sterile cotton swab, and wells were made aseptically using borer tool on MHA. Ten microliters of MB from each concentration was transferred into each well, and after overnight incubation at 37 °C, the inhibition halos were examined.

#### In vitro antibacterial photodynamic application on *P. aeruginosa*

For the *in vitro* aPDT of *P. aeruginosa,* the sample was divided into three corresponding groups: control/untreated (-MB-L), treated with laser without MB (-MB+L), and laser treated with MB (+MB+L). For laser irradiation without MB, a 100 μl aliquot was transferred directly into each well of a 96-well plate from a -20 °C stored bacterial strain in PBS suspension of the 0.5 McFarland standard (OD = 0.8, 10^8^ CFU/ml). Also, for aPDT treatment with methylene blue, 100 μl/ml of MB from each concentration (500, 250, 125, 62.5, 31.25, and 15.625 μg/ml) was mixed in 1 ml of bacterial suspension in Eppendorf tubes and incubated for 20 min at room temperature in the dark. After incubation, all samples were irradiated with and without methylene blue except the control group in 96-well plate at power densities of 300 mW/cm^2^ and 250 mW/cm^2^ for the total energy densities 18, 36, 54, 72, 90, and 109 J/cm^2^ and 15, 30, 45, 60, 75, and 90 J/cm^2^ using a 635-nm diode laser for exposure of 60, 120, 180, 240, 300, and 360 s. After photosensitization, the bacterial samples were spread on nutrient agar aseptically and incubated for 24 h at 37 °C. After incubation, the colonies were counted using the gold standard colony forming unit (CFU/ml) technique (Eq. 3) (Manzoor et al. 2022) to confirm the viability of the bacteria.

3 $$\frac{\textrm{CFU}}{\textrm{ml}}=\frac{\textrm{number}\ \textrm{of}\ \textrm{colonies}\times \textrm{dilution}\ \textrm{factor}\ \textrm{in}\ \textrm{total}}{\textrm{volume}\ \textrm{of}\ \textrm{culture}\ \textrm{plated}\ }$$

#### Optical density before and after aPDT treatment

It is common practice to determine the bacterial load in aqueous solutions using the optical density (OD) technique. However, the optical density (OD = 600 nm) of treated and untreated bacterial samples with aPDT at powers of 300 mW/cm^2^ and 250 mW/cm^2^ with MB concentrations for the selected exposure times and energy doses was measured by using a microplate spectrophotometer (INNOTM & INNO-MTM) at 600 nm. Each sample recorded three readings, and the average was used for the comparison research. Error bars have been added to the respective sections to highlight how the data varied from sample to sample.

#### Antibacterial PDT measurement with fluorescence spectroscopy

The FluoroMax-4 spectrofluorometer (HORIBA Scientific, Jobin Yvon, Germany) was used to record the emission spectra between 285 and 525 nm with a 270-nm excitation wavelength, operating with FluorEssence™ software version 3.5. The spectrometer incorporates a single position holder for sample measurement in a cuvette, a 150 W continuous wave (CW) Xenon discharge ozone-free arch lamp, and two monochromators for both emission and excitation sides. The increment resolution was reported at 1.00 nm, and the entrance and exit slit bandpass was reported at 5.00 nm. The fluorescence spectra of a 3-ml bacterial sample was acquired in a quartz cuvette with a 0.1-s integration time right after aPDT treatment at both power densities for the selected exposure time and dosages. Two spectra were recorded for each sample at each time.

#### Confocal laser scanning microscopy (CLSM) analysis

Confocal microscopic analysis was performed to evaluate the efficiency of MB-induced aPDT of *P. aeruginosa* by using a confocal laser scanning microscope (Zeiss LSM 510, Germany). The bacterial samples were excited by the 488-nm argon laser of 30 mW and the 543-nm HeNe laser of 1 mW power in CLSM. Confocal fluorescence photographs were observed of treated and untreated bacteria with a plan-apochromatic objective lens at 100x/1.40 Oil DIC M27 magnification with a 1.61 μsec Pixel Dwell and 900-ms scan time. The bacterial strain was irradiated with a 635-nm diode laser at selected powers, energy densities, and exposure times. After irradiation, the strain was mounted on glass slides and incubated in the dark at room temperature for drying. The CLSM was applied after washing the glass slides with autoclaved PBS to remove unattached bacterial cells and cover them with coverslips.

#### Statistical analysis

Treated and untreated groups were statistically analyzed using OriginLab and Microsoft Excel. All obtained results were compared with the control groups, and data taken in three readings were presented as mean ± standard deviation. Additionally, logarithmic values were assigned to data measured in colony-forming units.

## Results

### Antibiotic resistance confirmation of *P. aeruginosa*

**Fig. 1:**
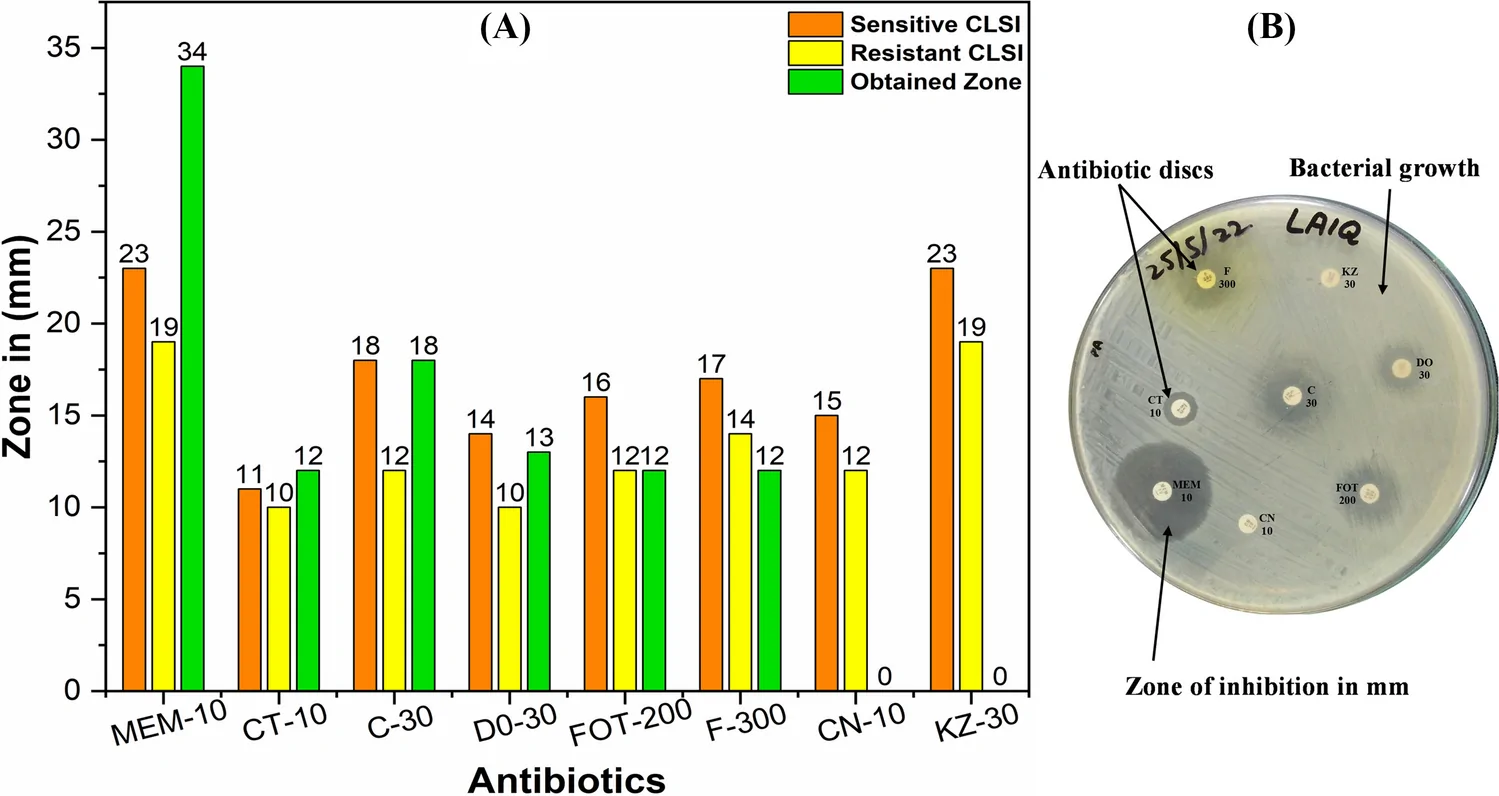
Antimicrobial susceptibility of *Pseudomonas aeruginosa*. **A** Comparison of antibiogram results with CLSI 2020. **B** Zone of inhibition measured in mm on MHA-containing plate

The diameter of ring-form inhibition zones was measured in mm using a digital Vernier caliper and compared to the values of CLSI 2020 standards (Fig. 1A, B). The clear inhibition zone around antibiotic discs represents bacterial susceptibility; the visible growth lawn and diameter of zones smaller than the standard reference values represent bacteria resistant to antibiotics. It is evident from Fig. 1 and Table 1 that *P. aeruginosa* is a multi-drug-resistant strain because it is resistant to four out of eight antibiotic classes and intermediate to a single one.

**Table 1:** Antibiotic susceptibility and resistance of *Pseudomonas aeruginosa* and comparison with the reference values of CLSI 2020

Antibiotics	Concentrations	S	R	Zone in mm	S/R/I
Meropenem (MEM)	10 μg	≥ 23	≤ 19	34	S
Colistin (CT)	10 μg	≥ 11	≤ 10	12	S
Chloramphenicol (C)	30 μg	≥ 18	≤ 12	18	S
Doxycycline (DO)	30 μg	≥ 14	≤ 10	13	I
Fosfomycin (FOT)	200 μg	≥ 16	≤ 12	12	R
Nitrofurantoin (F)	300 μg	≥ 17	≤ 14	12	R
Gentamicin (CN)	10 μg	≥ 15	≤ 12	0	R
Cefazolin (KZ)	30 μg	≥ 23	≤ 19	0	R

*mm* millimeter, *R* resistant, *S* sensitive, *I* intermediate

### Photodegradation of methylene blue

**Fig. 2:**
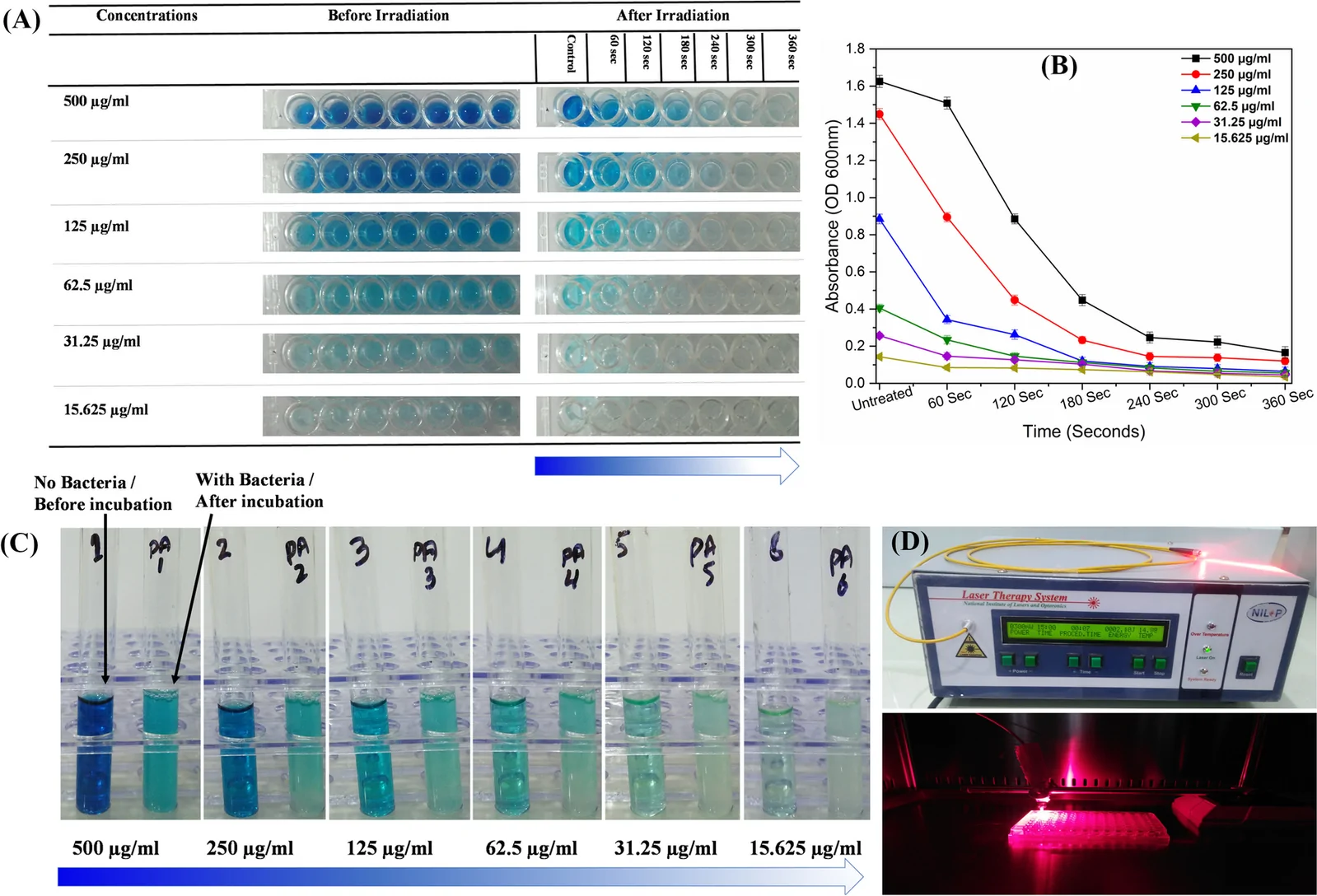
**A** Photobleaching of methylene blue concentration at a power of 300 mW/cm^2^ for selected irradiation times and dosages. **B** Optical density of control groups and photodegraded methylene blue at 600 nm. Mean ± standard deviation indicated by errors bar. **C** Methylene blue degradation before and after incubation with *P. aeruginosa.* **D** 635-nm diode laser therapy system developed by NILOP and their application inside a biosafety cabinet. μg/ml indicates methylene blue concentrations

Photodegradation is a normal phenomenon that occurs in typical compounds when they are exposed to light irradiation. The photobleaching experiment in the lab showed a high degradation activity of all methylene blue concentrations after exposure to different light doses for selected time durations at 300 mW/cm^2^ power (Table 2). Methylene blue concentrations of 500 μg/ml, 250 μg/ml, and 125 μg/ml changed from dark blue to light blue by attaining 16%, 35%, and 51.5% degradation after 60, 120, and 180 s of exposure, while 79%, 87.6%, and 90.5% of degradation occurred after 240, 300, and 360 s of exposure. All the other concentrations were degraded entirely and converted to a completely transparent color due to the minimal concentrations of MB (Fig. 2A).

**Table 2:** 635-nm diode laser set dosages for photodegradation of methylene blue at 300 mW/cm^2^

Time	60 s	120 s	180 s	240 s	300 s	360 s
Energy	18 J/cm^2^	36 J/cm^2^	54 J/cm^2^	72 J/cm^2^	90 J/cm^2^	108 J/cm^2^
Power	300 mW/cm^2^					

*s* seconds

The absorbance reduction after irradiation also confirmed the degradation of methylene blue by using a microplate spectrophotometer (INNOTM & INNO-MTM) after exposure to a diode laser (Fig. 2B). So, the higher the concentration of dye, the more exposure time and energy density are required.

### Methylene blue degradation using *P. aeruginosa*

The degradation of methylene blue before and after incubation with *P. aeruginosa* at temperature (37 °C) for overnight incubation and concentrations of MB are illustrated in Fig. 2C. A clear visual change in color from dark blue to transparent, compared with the control group, indicates that bacteria produced some chemicals and enzymes (Ikram et al. 2022) which cause the degradation of methylene blue. This demonstrates the potency of *Pseudomonas aeruginosa* with 90–99% of MB degradation. It is proved from the obtained results that methylene blue lost its potency as a photosensitizer after complete degradation by *Pseudomonas aeruginosa*. Hence, the higher the dye concentration, the more incubation time is required for its complete degradation.

### Efficiency of methylene blue in the dark and laser alone on bacterial growth

To study the antibacterial activity of methylene blue in the dark, all the concentrations of MB in the absence of laser irradiation (+MB-L) against *P. aeruginosa* were spread on nutrient agar plates and also on Mueller-Hinton agar through the well diffusion method. For overnight incubation at 37 °C, all plates were analyzed for bacterial load reduction.

**Fig. 3:**
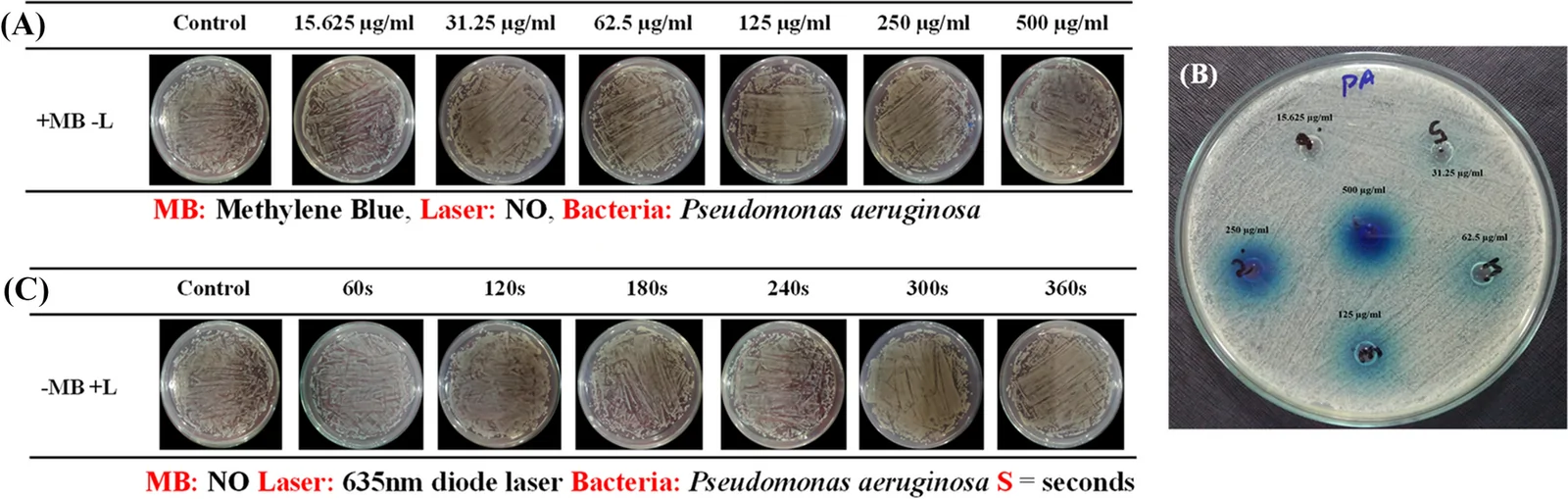
**A** Antibacterial activity of methylene blue using 15.625 μg/ml, 31.25 μg/ml, 62.5 μg/ml, 125 μg/ml, 250 μg/ml, and 500 μg/ml concentrations in the absence of light on *P. aeruginosa.* **B** Application of MB alone in well diffusion method on MHA-containing plate. **C** Application of 635-nm diode laser without methylene blue against *P. aeruginosa*. s, seconds. μg/ml indicates methylene blue concentrations

Still, according to Fig. 3A, no elimination was observed in viable bacterial growth on Petri plates, with no differences between the control and treated groups. Obviously, no clear zone of inhibition was observed on MHA through well diffusion except for the diffusion of methylene blue in wells and a clear bacterial lawn (Fig. 3B).

In another experiment for the efficiency of laser irradiation without methylene blue (-MB+L), a 635-nm diode laser developed by NILOP was used on *P. aeruginosa* at 300 mW/cm^2^ power for time exposures at 18, 36, 54, 72, 90, and 108 J/cm^2^ dosages. However, no reduction in bacterial load was observed, and the same number of viable colonies was present on Petri plates with no difference as compared with the control group (Fig. 3C). Hence, these results showed that methylene blue in the dark and light alone is ineffective on MDR *P. aeruginosa* and shows *no antibacterial activity*.

### Photodynamic inactivation of *P. aeruginosa*

**Fig. 4:**
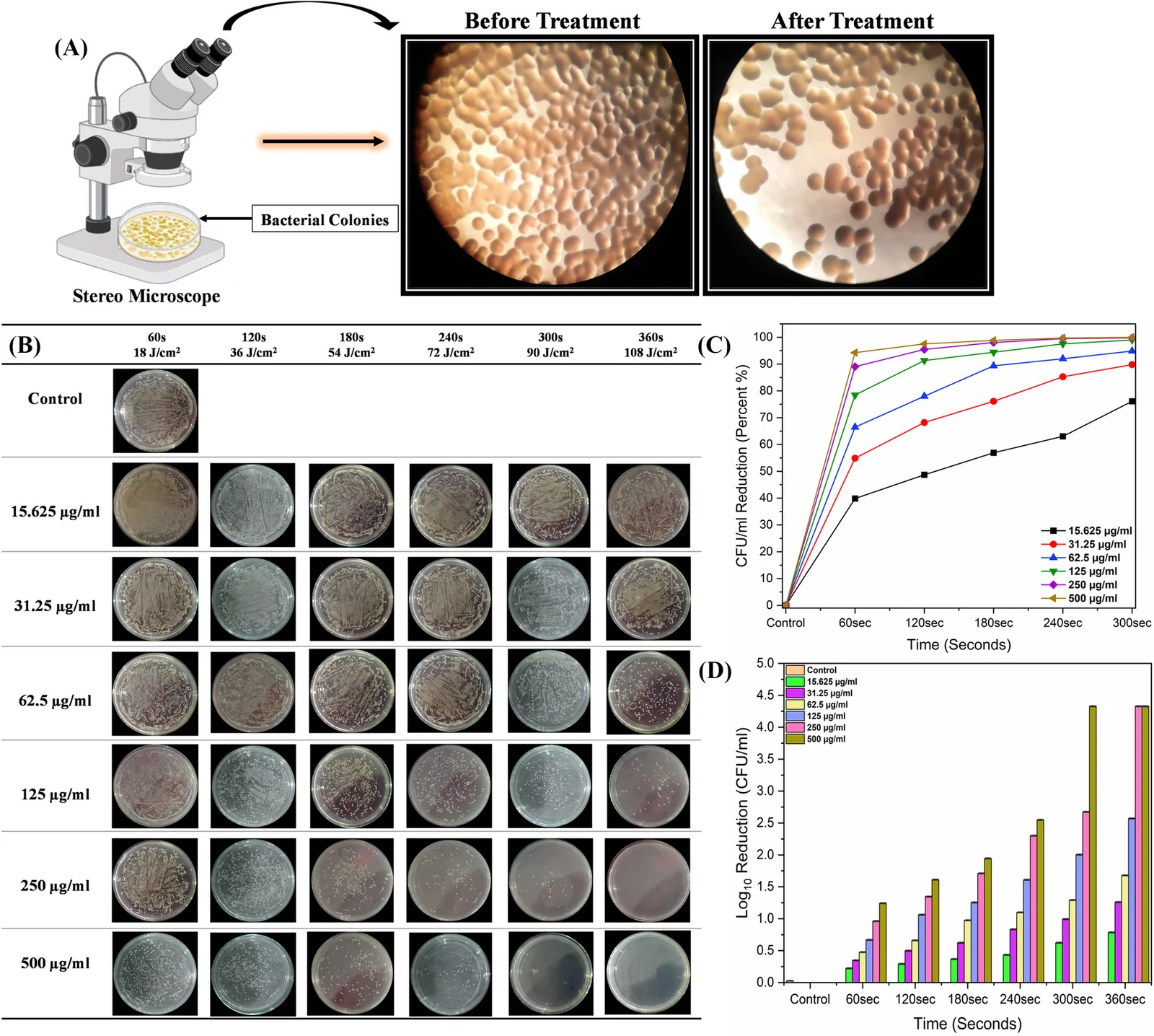
**A** *P. aeruginosa* colonies clearly seen under S/ST Stereo Microscope before and after treatment with MB and diode laser. **B** Photographs of *P. aeruginosa* colonies on nutrient agar plates after treatment with various concentrations of methylene blue in combination with 635-nm diode laser at a power of 300 mW/cm^2^ for exposures of 60, 120, 180, 240, 300, and 360 s and 18, 36, 54, 72, 90, and 108 J/cm^2^ of dosages. **C** Indicating a reduction of CFU/ml in percentage. **D** Log_10_ reduction of CFU/ml after treatment compared with the control group. The standard errors are very low and are presented as the mean ± standard deviation of each independent sample, which also demonstrates the logarithmic values in log_10_ CFU/ml reduction. s, seconds. μg/ml indicates methylene blue concentrations

Synergistically, methylene blue with a 635-nm diode laser system (+MB+L) was employed for the inactivation of *P. aeruginosa.* Consequently, a significant reduction in bacterial growth was confirmed under the S/ST Stereo Microscope (Kwun Tong, Kowloon, Hong Kong) (Fig. 4A). Indeed, the results were investigated by the standard gold method of colony-forming units (CFU/ml) before and after treatment, and the reduction in the viability of bacterial growth was compared with the control groups.

**Fig. 5:**
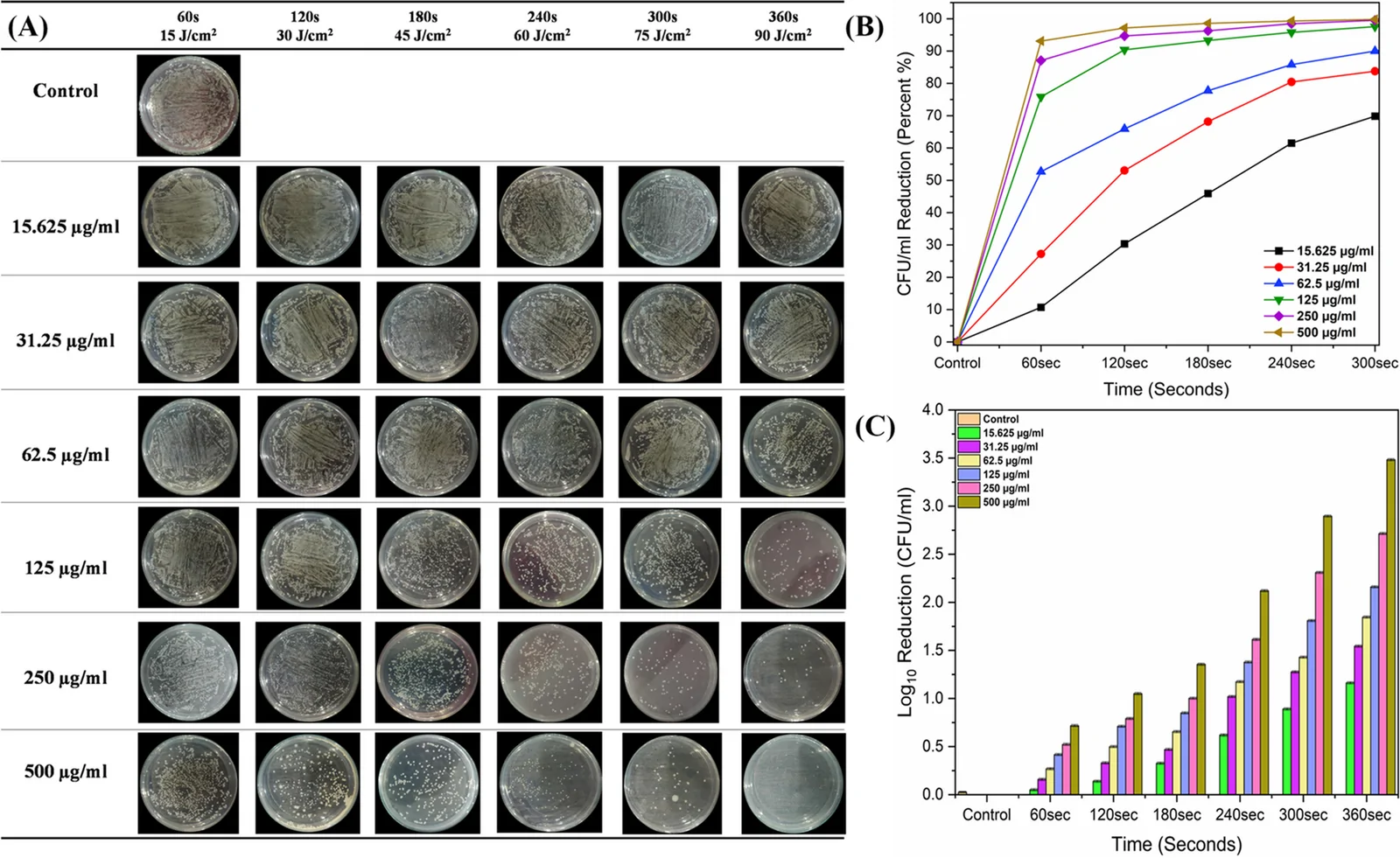
**A** Photographs of *P. aeruginosa* colonies on nutrient agar plates after treatment with various concentrations of methylene blue in combination with 635-nm diode laser at a power of 250 mW/cm^2^ for exposures of 60, 120, 180, 240, 300, and 360 s and 18, 36, 54, 72, 90, and 108 J/cm^2^ of dosages. **B** Indicating reduction of CFU/ml in percentage. **C** Log_10_ reduction of CFU/ml after treatment compared with the control group. The standard errors are very low and are presented as the mean ± standard deviation of each independent sample, which also demonstrates the logarithmic values in log_10_ CFU/ml reduction. s, seconds. μg/ml indicates methylene blue concentrations

For in vitro inactivation of *P. aeruginosa,* various concentrations of methylene blue from 15.625 to 500 μg/ml were used in conjugation with a 635-nm diode laser and illuminated in 96-well plate at both power densities for exposures of 60, 120, 180, 240, 300, and 300 s and energy densities of 18, 36, 54, 72, 90, and 108 J/cm^2^ (300 mW/cm^2^) and 15, 30, 45, 60, 75, and 90 J/cm^2^ (250 mW/cm^2^). As a result, a significant reduction in viable bacterial growth was observed compared to the treatment with methylene blue in the dark, diode laser alone, and control groups. All the results were calculated as a log_10_ CFU/ml reduction (Fig. 4 and Fig. 5).

In the case of photoinactivation at 300 mW/cm^2^, bacterial viability was reduced by 39.8%, 54.8%, 66.4%, 78.4%, 89%, and 94.2% (0.22 to 1.24 log_10_ reduction in CFU/ml) at 15.625 μg/ml, 31.25 μg/ml, 62.5 μg/ml, 125 μg/ml, 250 μg/ml, and 500 μg/ml MB concentrations after exposure for 60 s at energy density of 18 J/cm^2^ (Fig. 4C, D).

While as the exposure time for treatment and energy density increased, the viability was reduced, respectively, from 48.7 to 97.5% (0.28 to 1.60 log_10_) for 120 s at 36 J/cm^2^, 56.9 to 98.8% (0.36 to 1.94 log_10_) for 180 s at 54 J/cm^2^, 63 to 99.7% (0.43 to 2.54 log_10_) for 240 s at 72 J/cm^2^, and from 76.1 to 99.99% (0.62 to 4.32 log_10_ reduction in CFU/ml) for 300 s at 90 J/cm^2^. Moreover, aPDT at 108 J/cm^2^ for 360 s of exposure, the bacterial growth was reduced by 83.5%, 94.4%, 97.8%, 99.7%, 99.99%, and 99.99%, with a 0.78 to 4.32 log_10_ reduction in CFU/ml (Fig. 4B–D). According to Fig. 4B, the same number of colonies was inhibited by MB-mediated aPDT at 250 μg/ml and 500 μg/ml MB concentrations for 360 s of exposure at energy of 108 J/cm^2^, while also after treatment for 300 s at 500 μg/ml (90 J/cm^2^).

On the other hand, it is evident that a reduction from 0.04 to 3.48 log_10_ in CFU/ml of *P. aeruginosa* was observed with the treatment at a power of 250 mW/cm^2^ as compared to the control group and treatment with MB in the dark and laser alone (Fig. 5A–C).

As displayed in the plate photographs in Fig. 5A, the bacterial colonies decreased by 10.6%, 27.2%, 52.6%, 75.8%, 87%, and 93.1%, with a reduction from 0.04 to 1.16 log_10_ in CFU/ml at MB concentrations of 15.625 μg/ml, 31.25 μg/ml, 62.5 μg/ml, 125 μg/ml, 250 μg/ml, and 500 μg/ml after 60 s of exposure at 15 J/cm^2^ density of energy (Fig. 5B, C), while the killing rate of colonies increased from 30.3 to 97.1% (0.15–1.54 log_10_ reduction in CFU/ml) after 120 s at 30 J/cm^2^, 45.9 to 98.5% (0.26–1.84 log_10_) after 180 s at 45 J/cm^2^, 61.5 to 99.3% (0.41–2.15 log_10_) after 240 s at 60 J/cm^2^, and 69.8 to 99.8% (0.52–2.71 log_10_) after 300 s at 75 J/cm^2^ as a consequence of the rise in the time exposure to irradiation and density of energy.

In addition, the increased inhibition impact was reported by 80.7%, 91%, 95.5%, 99.2%, 99.8%, and 99.97%, with a reduction in CFU/ml from 0.71 to 3.48 log_10_ at 90 J/cm^2^ after irradiation exposure for 360 s (Fig. 5A–C). However, when *P. aeruginosa was* treated with MB-based aPDT at a power of 300 mW/cm^2^, it showed the maximum reduction in comparison treatment at a power of 250 mW/cm^2^ (Fig. 4 and Fig. 5). The data indicated that *P. aeruginosa* is more susceptible to 300 mW/cm^2^ at 90 and 108 J/cm^2^ for 240, 300, and 360 s of exposure than 250 mW/cm^2^ laser power.

### Optical density-based analysis

**Fig. 6:**
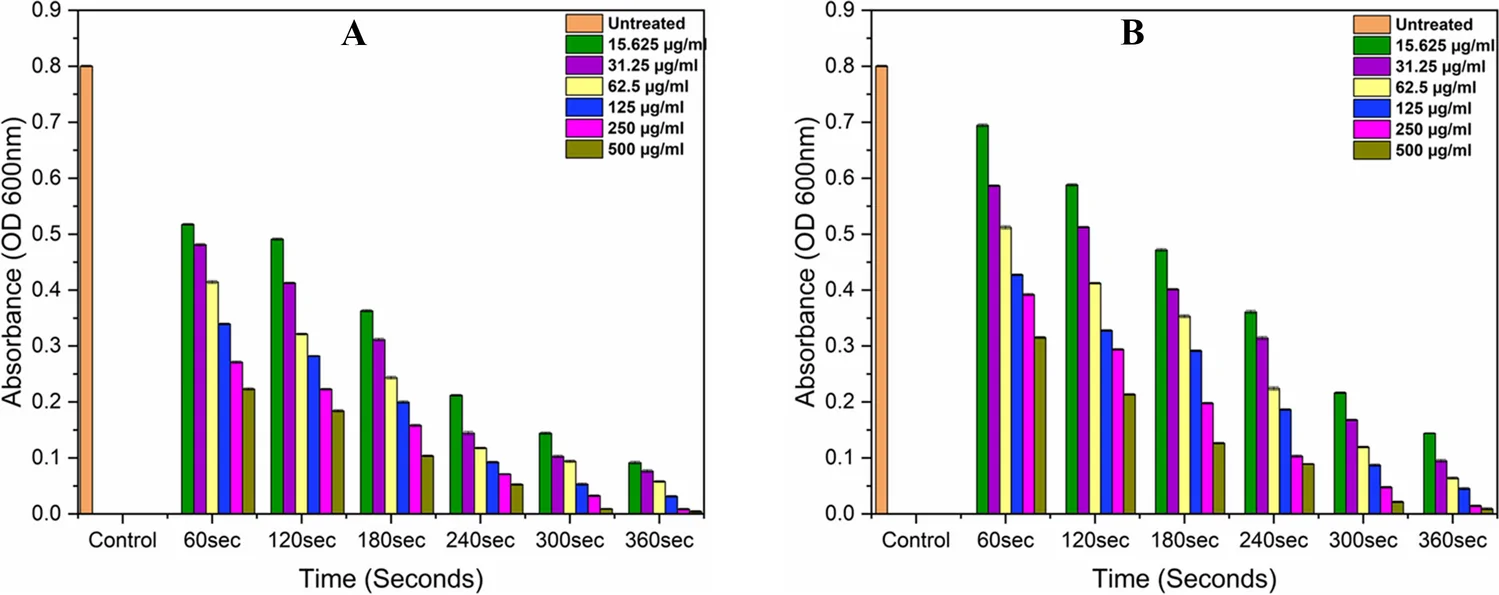
Optical density of *P. aeruginosa* was measured at a wavelength of 600 nm before and after treatment with aPDT at powers **A** 300 mW/cm^2^ and **B** 250 mW/cm^2^. The standard errors are very low and are presented as the mean ± standard deviation of each independent sample. μg/ml indicates methylene blue concentrations

The measurement of OD is a rapid, cheap, and frequently used technique for determining the quantity of viable bacterial cells in the aqueous solution at the 600-nm wavelength; at this wavelength, the light scatters and measures the turbidity in the solution, which indicates the increase and decrease in the level of bacteria (Beal et al. 2020; Imtiaz et al. 2023). Figure 6A and B demonstrate the optical density of *P. aeruginosa*, measured at 600 nm before and after treatment with various concentrations of methylene blue and a 635-nm diode laser.

As the irradiation time and dosages increase, the solution turbidity and OD gradually decrease compared to the control group which indicates the highest level of bacterial growth. After 360 s of exposure and irradiation with powers of 360 mW/cm^2^ (108 J/cm^2^) and 250 mW/cm^2^ (90 J/cm^2^), in the last sample, complete transparency was observed. The obtained results of optical densities as shown in Fig. 6A and B correlate with Fig. 4 and Fig. 5, which illustrate a maximum decrease in bacterial colonies on nutrient agar plates after aPDT treatment.

### Fluorescence spectroscopy for the study of *P. aeruginosa* inactivation

**Fig. 7:**
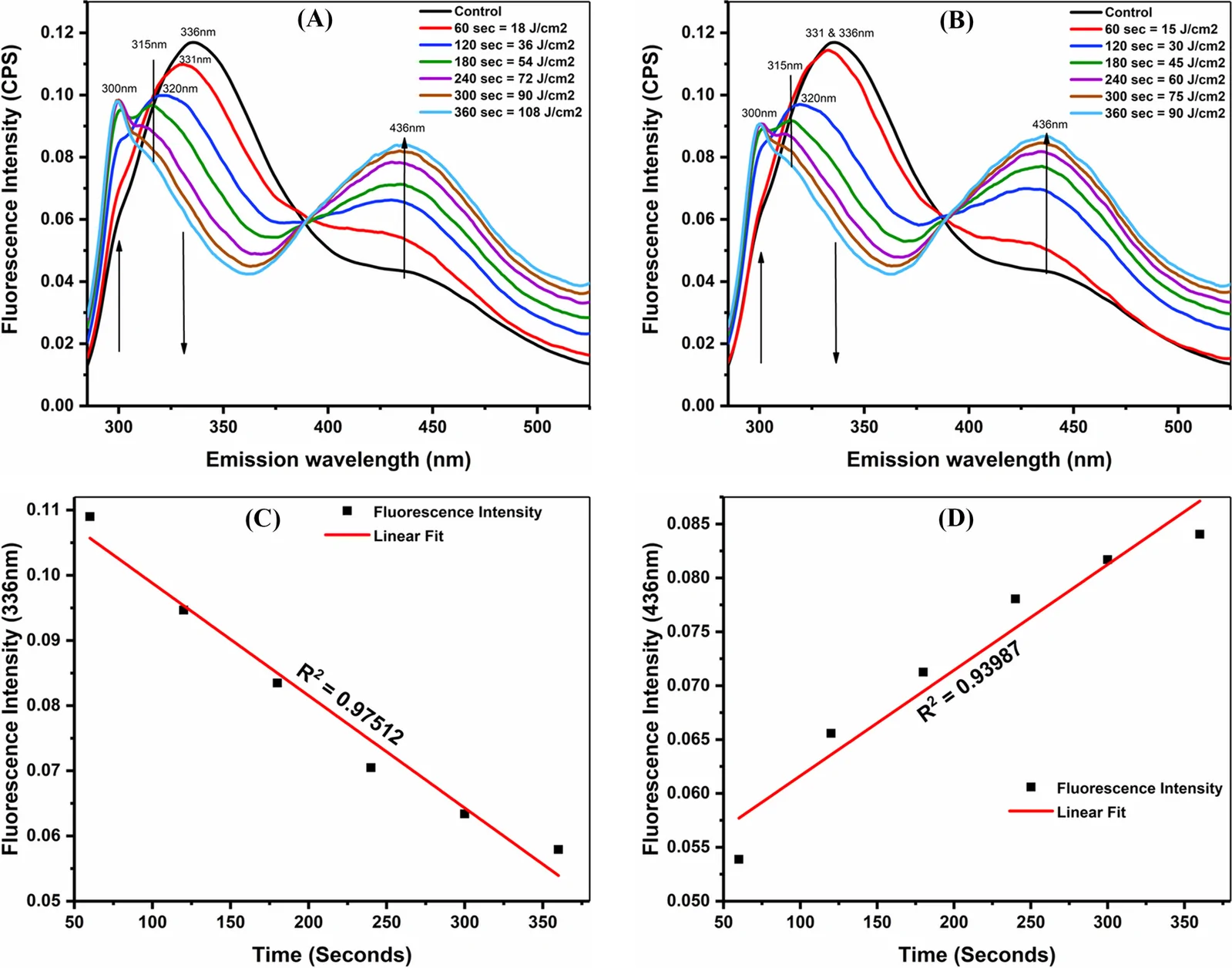
Spectra of fluorescence spectroscopy were recorded at excitation wavelength 270 nm and emission wavelength 285 to 525 nm after treatment with various concentrations of methylene blue for specific time exposure at powers **A** 300 mW/cm^2^ (18, 36, 54, 72, 90, and 109 J/cm^2^) and **B** 250 mW/cm^2^ (15, 30, 45, 60, 75, and 90 J/cm^2^). **C** Linear relationship/calibration between fluorescence intensity at 336 nm and **D** 436 nm and exposure time to determine the gradual decrease and increase in fluorescence intensity after treatment

Figure 7A and B demonstrate the fluorescence spectroscopy of *P. aeruginosa* before and after aPDT treatment at powers of 300 mW/cm^2^ and 250 mW/cm^2^ for selected time exposures and laser dosages. Fluorescence spectroscopy is an advanced, fast, and authentic technique for detecting viable bacteria in aqueous solutions through monitoring the fluorophores in microorganisms (Ammor 2007). The fluorescence emission spectra of bacteria were recorded between 285 and 525 nm, with excitation at 270 nm. Figure 7A and B show the broad spectral peak at 336 nm, which represents the signature for tryptophane molecules, that was later blue shifted from 336 to 309 nm after treatment towards the left side. Consequently, after the elimination of bacterial viability and degradation of methylene blue, a new spectral peak appeared after 120 s of exposure (30 J/cm^2^ and 36 J/cm^2^) at 300 nm, which indicates the water Raman scattering (Imtiaz et al. 2023). A variation in the reduction of bacteria after treatment with both power densities compared to the control group was also observed and was confirmed by colony-forming unit per milliliter and optical density (Fig. 4, Fig. 5, and Fig. 6). Tryptophane is a highly fluorescent amino acid or fluorophore that indicates bacterial viability in aqueous solutions, which show emission spectra from 305 to 400 nm with excitation at 270 nm (Ammor 2007; Du et al. 2022). Moreover, a new fluorescence emission spectral band appeared at 436 nm, which could be attributed to the purity of the aqueous solution after a reduction in the quantity of bacterial cells.

Figure 7C and D depict the linear fit relationship between the fluorescence intensity at 336 nm and 436 nm and the exposure time to aPDT illumination with a linear coefficient of 0.975 and 0.939. In fact, it indicates the sensitivity of bacteria to the exposure time and dosages of aPDT. The new fluorescence band at 436 nm was gradually increased as compared to the fluorescence band at 336 nm, which was gradually decreased after the rise in exposure time and dosages of both power densities.

### Confocal microscopic analysis

**Fig. 8:**
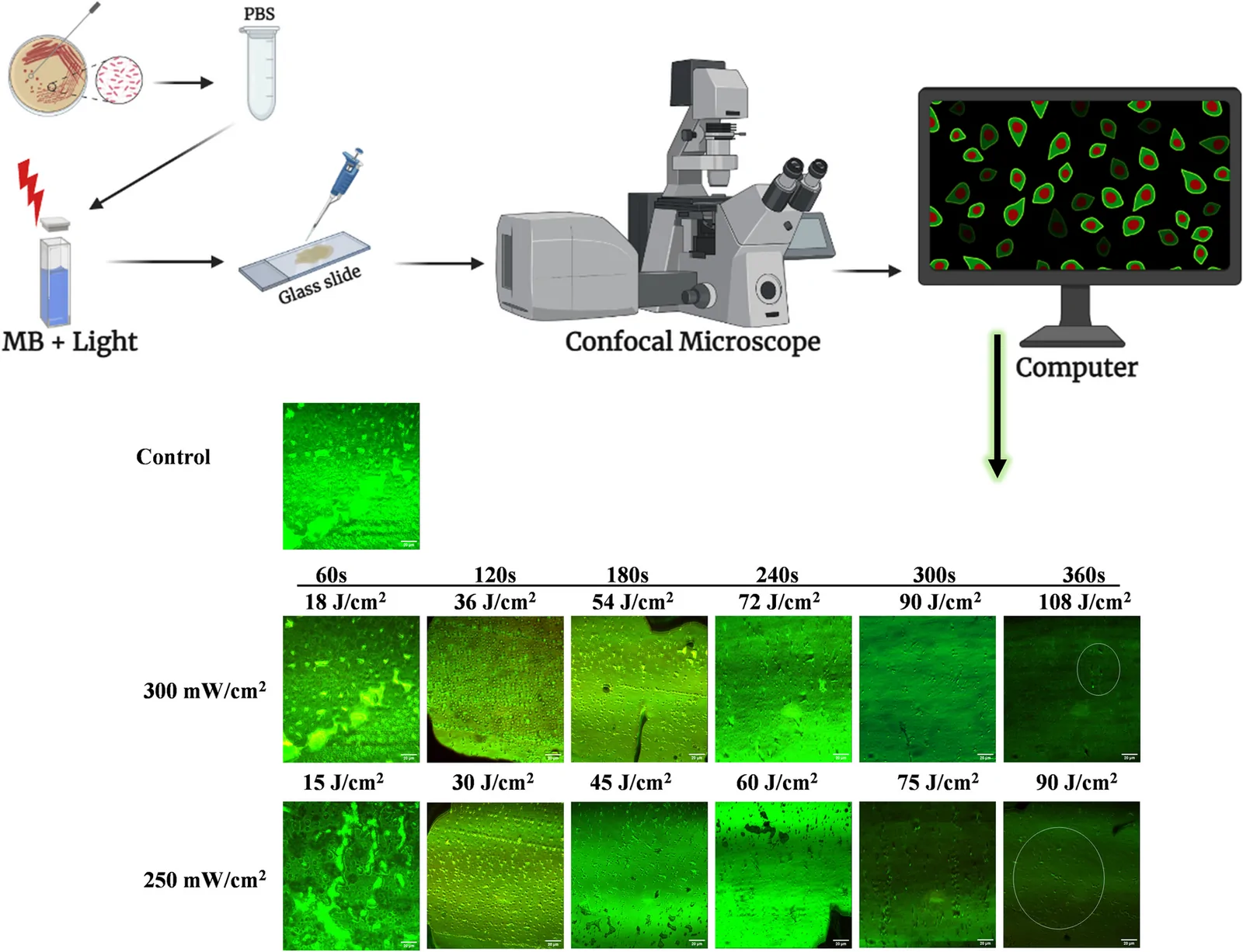
Confocal laser scanning microscopy (CLSM) images of *P. aeruginosa. The dense* green lawn, fluorescent colony-like structures represent the live cells, while the non-fluorescent empty places in the lawn indicate the inhibition of bacterial load after treatment with aPDT at powers of 300 mW/cm^2^ and 250 mW/cm^2^. The circles in the last treated samples indicate few low-fluorescent live colonies after treatment, while only four colonies remain live at 108 J/cm^2^ as compared to 90 J/cm^2^. s, seconds

*P. aeruginosa* morphology was visualized in images of confocal laser scanning microscopy before and after treatment with aPDT at powers of 300 mW/cm^2^ and 250 mW/cm^2^ in combination with methylene blue for time exposure from 60 to 360 s and energy densities (Fig. 8). The intense and dense fluorescent green mat of colonies in the control group and at the start of the treated sample photographs for 60 and 120 s shows maximum viability in bacterial cells.

According to Fig. 8, a significant and higher reduction was observed later after treatment with 54 to 108 J/cm^2^ and 45 to 90 J/cm^2^ of both power densities for 180 to 360 s of exposure, which indicates the photo-induced destruction in the growth of bacterial cells as compared to photographs of the control group.

## Discussion

Worldwide antimicrobial resistance and infectious diseases caused by pathogenic bacteria are serious public health concerns. In fact, most conventional antimicrobial drugs remain ineffective against many bacterial pathogens, which cause serious, long-lasting infections, disabilities, and mortalities (Parasuraman et al. 2020). Therefore, developing new strategies to tackle microorganisms resistant to many drugs has become more crucial than ever. As a novel strategy to effectively eradicate drug-resistant pathogens, antimicrobial photodynamic therapy (aPDT) has recently drawn a lot of interest, and it is also known that aPDT repetition does not result in bacterial resistance. So, by exposing photosensitizers to a specific wavelength of light, aPDT has a broad-spectrum activity that disrupts both resistant and susceptible microbial cells by producing molecular oxygen (Motallebi et al. 2020; Sarker et al. 2021).

Due to the distinct structure of the cell wall, *P. aeruginosa* is one of the most difficult forms of gram-negative bacteria to eradicate (Kim and Lim 2020). It is obvious that methylene blue is a cationic photosensitizer that has the potency to inhibit both gram-positive and gram-negative bacteria. Particularly due to the positive charge on methylene blue, it can easily bind to the negative charge (lipopolysaccharide) LPS of gram-negative bacteria to pass through their cell wall, while gram-positive bacteria have a porous peptidoglycan membrane layer, which makes it easier for MB to penetrate (Gollmer et al. 2015; Motallebi et al. 2020). In the proposed study, we investigate the potency of various concentrations of methylene blue against multi-drug-resistant *P. aeruginosa* by employing a 635-nm red-spectrum diode laser at powers of 300 mW/cm^2^ and 250 mW/cm^2^ for specific times of exposures.

Before the MB-mediated aPDT, we examined the efficacy of our selected 635-nm diode laser for the photodegradation of methylene blue at a power of 300 mW/cm^2^ without any catalyst addition. The result showed a maximum color change of MB from dark blue to transparent form, which resulted in dye degradation of 16% for 18 J/cm^2^ (60 s), 35% for 36 J/cm^2^ (120 s), and 51.5% for 54 J/cm^2^ (180 s). However, the highest degradation (79%, 87.6%, and 90.5%) was reported for 72, 90, and 108 J/cm^2^ after 240, 300, and 360 s of exposure. Rather et al. (2017) evaluated the photodegradation of methylene blue by using TiO_2_ (P25) in combination with Cu, Au, and Ag for 60 min for visible light radiation. They observed complete dye degradation after the addition of catalyst to TiO_2_ as compared to TiO_2_ (P25) alone, which shows a minimum range of degradation. In another study, Ren et al. (2015) used a 100-mW/cm^2^ AM 1.5 G Xenon lamp in combination with four different samples of TiO_2_ as a catalyst, and degradation of methylene blue from 23.5 to 84.3% was observed in the first three samples after 40 and 50 min of exposure, while sample 4 showed about 97.2% degradation after 20 min of irradiation. Moreover, Kalaycıoğlu et al. (2023) used UV-A and sunlight for the degradation of MB, which showed 90% and 50% potency after 90 min of irradiation.

According to the literature, *P. aeruginosa* is a pathogenic strain and has a high potency to degrade methylene blue under specific time durations and environmental conditions (Ariffin and Anuar 2022). Therefore, we also investigated the biodegradation of methylene blue at various concentrations from 500 to 15.625 μg/ml by using *P. aeruginosa* in the lab. Consequently, after overnight incubation at the optimum 37 °C temperature, a significant decolorization from 90 to 95% was reported from dark blue to a transparent color, representing the efficiency of *P. aeruginosa* in dye degradation. Eslami et al. (2016) isolated a bacterial strain of *P. aeruginosa* from contaminated soil and used it for the removal of methylene blue from an aqueous solution, which showed high potency in the degradation of MB from 82.2 to 97.8% at the best concentration of 200 mg/ml, while the degradation efficiency was decreased to 43.8% after an increase in the concentration of methylene blue to 1000 mg/ml. Furthermore, in another study, 10.5% of MB degradation was observed by *P. aeruginosa* at an optimum temperature of 35 °C after 8 days of incubation (Ariffin and Anuar 2022).

Next, various concentrations of methylene blue in the dark and a 635-nm diode laser without methylene blue were used against *P. aeruginosa* for specific time exposure to irradiation at a power density of 300 mW/cm^2^, but in both methods, no reduction of bacterial colonies was observed. As discussed in previous studies, methylene blue in the dark and laser alone are ineffective against certain bacterial strains. In another study, methylene blue alone and in addition with AgNPs in the absence of irradiation for 2 h of incubation did not show any reduction in bacterial growth (Vecchio et al. 2015). Even Sarker et al. did not observe any reduction in bacterial colonies after treatment with methylene blue alone and with the addition of 10% ethanol without irradiation (Ammor 2007). Kim et al. (Du et al. 2022) and Wardlaw et al. (Kalaycıoğlu et al. 2023) employed the 660-nm and 635-nm diode lasers against *P. aeruginosa* for exposures of 1 to 6 h, but at last, they also did not observe a significant reduction in the growth of bacteria.

Additionally, the laser alone at a power intensity of 2.5 W or 1 W has not successfully limited the number of bacterial colonies after 10 s of irradiation (Caccianiga et al. 2012). However, many studies proved that methylene blue-mediated antimicrobial photodynamic therapy (aPDT) showed maximum and significant bactericidal activity as compared to methylene blue in dark and laser light alone (Imtiaz et al. 2023). In addition, in our study, methylene blue at different concentrations ranging from 500 to 15.625 μg/ml with a 635-nm diode laser at powers of 300 mW/cm^2^ and 250 mW/cm^2^ after 60 to 360 s of irradiation exhibited significant antibacterial activity as compared to the control group, laser alone, and methylene blue in the dark.

The inactivation of bacteria is not only dependent on the efficiency of laser light, but there are some common factors such as time of incubation, the concentration of specific photosensitizers, the intensity of power, energy density, exposure time, laser light wavelength, and a distance of the light from the samples that influence the viability of bacterial strains (Calin et al. 2013; Parasuraman et al. 2020). Fluorescence spectroscopy and optical density were employed for the measurement of bacterial growth; also CLSM photographs showed the photoinactivation of *P. aeruginosa* after treatment with methylene blue at an appropriate wavelength of diode laser (Parasuraman et al. 2020), which was further confirmed by calculating the colony-forming units and log_10_ reduction in bacterial colonies. In addition, colonies of *P. aeruginosa* were reduced by 1.94 log_10_ after 54 J/cm^2^ for 180 s, 2.54 log_10_ after 72 J/cm^2^ for 240 s, and 4.32 log_10_ after 90 J/cm^2^ irradiation for 300 s at a power density of 300 mW/cm^2^, while at an intensity of 250 mW/cm^2^, the reduction was observed by 1.84 log_10_, 2.15 log_10_, and 2.71 log_10_ at 45, 60, and 75 J/cm^2^ after the irradiation of 500 μg/ml of methylene blue with a 635-nm diode laser.

Furthermore, Yang et al. (2019) observed a 3 log_10_ decrease at 20 J/m^2^ and a 5.5 log_10_ decrease at 30 J/cm^2^ in the viability of *P. aeruginosa* after irradiation of 250 μM, 750 μM, and 1 mM of methylene blue with a 660-nm diode laser at a power of 300 mW/cm^2^ for exposures of 8 min and 12 min at planktonic state, while a 2.5–3 log_10_ reduction was observed in colonies of *P. aeruginosa* in the biofilm state. Moreover, the biofilm of *P. aeruginosa* was reduced by 6 log_10_ and 7 log_10_ after the addition of hydrogen peroxide to MD-based aPDT at 20 J/cm^2^ and 30 J/cm^2^, respectively. Similarly, 100 μg/ml, 300 μg/ml, and 500 μg/ml concentrations of methylene blue reduced the growth of *P. aeruginosa* by 5.0 log_10_ after treatment with 70 mW 660-nm LED at energy densities of 10 J/cm^2^ and 25 J/cm^2^ for 6 min and 40 s of exposure (Pereira et al. 2018). Besides, a significant reduction was reported in bacterial viability in both planktonic and biofilm states after the addition of additives such as viscous buffers, EDTA, ethanol, and hydrogen peroxide because these additives worked as catalysts that enhanced the antibacterial activity of methylene blue-based antimicrobial photodynamic therapy (Street et al. 2009; Biel et al. 2013; Prochnow et al. 2016).

The advantages of photodynamic therapy over typical chemical antimicrobial agents are as follows: first, it eliminates a broad range of bacteria; second, it quickly kills microorganisms, usually within a few seconds or minutes; and third, resistance is improbable to the PDT treatment (Wilson 2004). Moreover, antimicrobial photodynamic therapy (aPDT) has several other important benefits, including being less expensive than conventional chemical therapy, friendly to the environment, and having high safety in a variety of applications (Cantelli et al. 2020). As compared to chemical therapy, the aPDT effectively eliminates a broad range of microbes. They have effective phototoxicity to kill both antimicrobial-resistant and wild-type bacterial and other microbial strains. In addition, they have a low mutational potential and are highly selective in eliminating pathogenic strains. It is very specific and efficient in terms of time and space selectivity and produce highly toxic ROS and oxygen for microbe eradication (Anas et al. 2021). If PDT has a lot of benefits, there are some disadvantages as well. Because of the broad-spectrum effects of ROS (reactive oxygen species) produced during PDT treatment, it has the potential to eliminate both harmful (pathogenic) and beneficial microorganisms. For instance, if ROS concentrations are higher than what the host can tolerate, in that situation, the host cells might be inactivated due to the unintended effects. The reaction may be managed using the most recent technological advancements by regulating the concentrations of photosensitizers, exposure times of light and chemicals, and intensity of light (Montanha et al. 2017). According to reports, *Pseudomonas aeruginosa* is significantly killed by aPDT. Also, Alam et al. (2019) described that the synergism of ampicillin and hypericin with the conjugation of orange light successfully eradicated *Pseudomonas aeruginosa*. However, in this study, we did not use any type of catalyst for the enhancement of bacterial inhibition, but the present study exhibited the antibacterial potency of the photosensitizer methylene blue in aPDT as compared in the dark against multi-drug-resistant *P. aeruginosa,* which is involved in severe skin infections.

In conclusion, the study demonstrates the efficiency of novel antimicrobial photodynamic therapy using methylene blue at various concentrations against *P. aeruginosa*. However, laser illumination alone and methylene blue in the dark did not express a significant difference as compared to the control group, while treatment with methylene blue-based aPDT exhibited a maximum of 3.48 log_10_ and 4.32 log_10_ reduction at 90 J/cm^2^ and 108 J/cm^2^, respectively. Therefore, this *in vitro* activity of MB-based aPDT proved that it could be the best possible alternative for eliminating gram-negative pathogenic bacteria. The study further supports laser-based noninvasive therapy for the treatment of bacterial-infected skin diseases. In future clinical experiments, the best-selected laser power, dosages, and concentrations of methylene blue will be applied using cell lines and mice models to demonstrate the effectiveness of novel aPDT treatment for severe wounds and skin infections.

## Supplementary information

ESM 1 (PDF 255 kb)

## Data Availability

Data sharing is not applicable to this article as no datasets were generated or analyzed during the current study.
