# Supplementary material for: In vitro study: methylene blue-based antibacterial photodynamic inactivation of Pseudomonas aeruginosa
Source: Appl Microbiol Biotechnol. 2024 Jan 23;108:169. doi: 10.1007/s00253-024-13009-5 (PMC10806092; doi:10.1007/s00253-024-13009-5)
Supplement: Supplementary file 1 [file 253_2024_13009_MOESM1_ESM.pdf]

Journal: Applied Microbiology and Biotechnology

In vitro study: methylene blue-based antibacterial photodynamic inactivation of *Pseudomonas aeruginosa*

Laiq Zada[1,2], Shahzad Anwar[2*], Sana Imtiaz[2], Muhammad Saleem[2], Aamer Ali Shah[1*]

[1]Department of Microbiology, Quaid-i-Azam University, 45320, Islamabad, Pakistan.
[2]National Institute of Lasers and Optronics College, Pakistan Institute of Engineering and Applied Sciences, Nilore, Islamabad 45650, Pakistan.

*Corresponding authors

Shahzad Anwar, Ph.D.: Agri& Biophotonics Laboratory, National Institutes of Lasers and Optronics, Islamabad, Pakistan.

Email: Shahzadanwar112@yahoo.com

Aamer Ali Shah, Ph.D.: Applied Environmental and Geo-Microbiology Lab, Department of Microbiology, Quaid-i-Azam University, Islamabad, Pakistan.

Email: alishah@qau.edu.pk

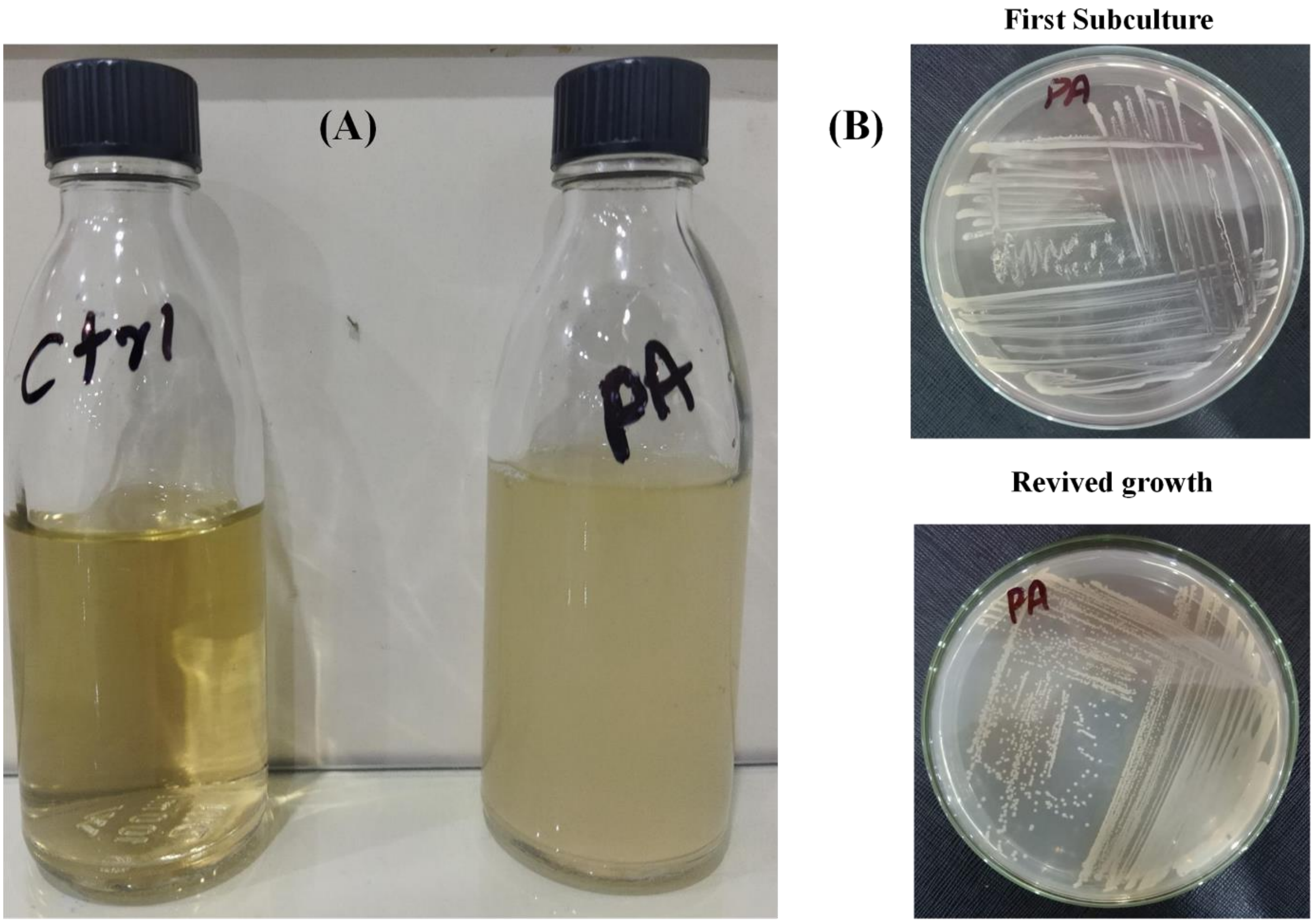

Figure S1: Preparation and purification of bacterial strain. (a) Inoculation of *Pseudomonas aeruginosa* in nutrient broth. **(b)** Revival of bacterial strain on nutrient agar.

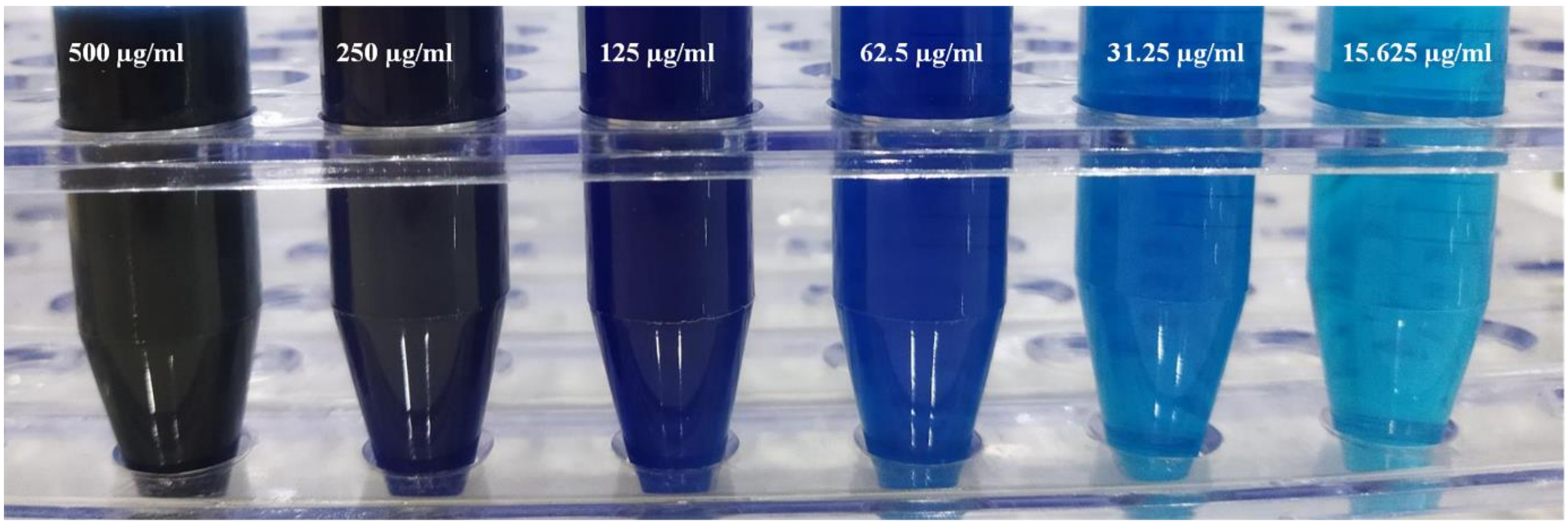

Figure S2: Methylene blue preparation from a stock solution in µg/ml concentrations for aPDT application
